# Metabolite Profiling, through LC-ESI/LTQOrbitrap/MS Analysis, of Antioxidant Extracts from *Physalis alkekengi* L.

**DOI:** 10.3390/antiox12122101

**Published:** 2023-12-12

**Authors:** Maria Assunta Crescenzi, Gabriele Serreli, Monica Deiana, Carlo I. G. Tuberoso, Paola Montoro, Sonia Piacente

**Affiliations:** 1 Department of Pharmacy, University of the Study of Salerno, Via Giovanni Paolo II 132, 84084 Fisciano, Italy; mcrescenzi@unisa.it (M.A.C.); piacente@unisa.it (S.P.); 2 Unit of Experimental Pathology, Department of Biomedical Sciences, University of Cagliari, Cittadella Universitaria SS 554, 09042 Monserrato, Italy; gabriele.serreli@unica.it (G.S.); mdeiana@unica.it (M.D.); 3 Department of Life and Environmental Sciences, University of Cagliari, Via Ospedale 72, 09124 Cagliari, Italy; tuberoso@unica.it

**Keywords:** LC-ESI/LTQOrbitrap MS, *Physalis alkekengi* L., antiradical scavenging activity, metabolic profiling

## Abstract

Due to the increasing use of *Physalis alkekengi* L. as a food supplement and starting material for tea preparation, a comprehensive analysis of green extracts was performed. Two different extraction methods were applied to yellow *Physalis alkekengi* L. fruit and calyx and compared: hydroalcoholic extraction and decoction. Characterization of the metabolome of the calyx and fruit of yellow *Physalis alkekengi* L. was performed by LC-ESI/LTQOrbitrap/MS followed by LC-ESI/LTQOrbitrap/MS/MS to identify 58 phytocompounds using the two different extraction techniques. Subsequently, through preliminary spectrophotometric assays followed by cell studies, the antioxidant activity of the different *Physalis alkekengi* L. extracts were evaluated. It was found that *Physalis alkekengi* L. extracts are a good source of metabolites such as flavonoids, organic acids, phenylpropanoids, physalins and carotenoids, with various biological activities, in particular, antioxidant activity capable of reducing the production of free radicals in intestinal Caco-2 cells. For the first time, an integrated approach (metabolomics approach and antioxidant evaluation) was applied to the study of *Physalis alkekengi* green extracts and decoctions, the green extraction method mostly used in herbal preparations. An interesting finding was the high antioxidant activity shown by these extracts.

## 1. Introduction

*Physalis alkekengi* L. (family Solanaceae) is a plant native to Asia and southern Europe. It is also called Chinese lantern, Japanese lantern, bladder cherry, and winter cherry. It is now spread all over the world [1].

It is characterized by an upright, tufted growth habit and can reach a height of up to 60 cm when fully grown. The light green leaves are alternate, oval, and slightly hairy. Between July and August (in the northern hemisphere), usually from the second year after planting, the plant bears fruit filled with berries about 17 mm in diameter, which are covered in a papery calyx; they can be eaten both cooked and raw, and are the only edible part of the plant [2].

The formation of the fruit is fascinating: the calyx swells to a length of 5–7 cm, then swells like a balloon of parchment paper and changes color, turning from the initial green to a bright red of autumn. If the fruit remains on the plant during the winter season, the husk slowly disintegrates, and only the fibrous scaffold remains intertwined with the berry inside [1].

*Physalis alkekengi* L. is rich in steroids, flavonoids, terpenoids, alkaloids, phenylpropanoids, phenolic acids, etc. As for many other species of the genus *Physalis*, it contains a wide variety of physalins [3,4].

It has medicinal properties, so has been cultivated since ancient times [5,6]. It must be noted that it is a toxic plant in all its parts except the berry, which is very juicy, and has a fresh and somewhat bitter taste. Bitterness tends to decrease if the fruit is frozen [7]. The berries contain large amounts of vitamin C, citric acid, tannin, and sugar [2,8]. Among its therapeutic properties, its use in the treatment of kidney and bladder stones and as a diuretic stand out.

The Chinese lantern has many biological activities such as anti-inflammatory, antibacterial, antiseptic, sedative, laxative, diuretic, hypoglycemic, and spasmolytic, which is why it is widely used in traditional medicine. In Chinese medicine, *Physalis alkekengi* L. is a remedy for several diseases [9].

Extracts and complex fractions isolated from *Physalis* plants are known in the literature to show biological and pharmacological activities, and most are associated with the presence of the above-mentioned secondary metabolites. The total data disclose the potential of *Physalis* spp. as highly functional foods, profitable crops, and as sources of valuable secondary metabolites, novel medicine and cosmetics [8].

It has been shown in the literature that the phenolic compounds identified in *Physalis alkekengi* L. are able to inhibit the growth of breast and colon cancer cells [10]. In addition, the fruit peel extract showed anti-inflammatory activity by relieving inflammation in mice with inflammatory bowel disease [11]. The fruit contains compounds called withanolides that may have anti-inflammatory effects, potentially protecting against colon cancer [12]. *Physalis alkekengi* L. is a good source of antioxidants partly due to its high vitamin C content, but also due to the presence of bioactive compounds such as physalines and flavonoids [10]. For this reason, its intake can help the body counteract the activity of free radicals. These compounds, which are a by-product of human metabolism, are often the cause of serious diseases such as cancer. Etzbach et al. demonstrated that the skin of the berries (332.00 µg/g dw) has almost three times the amount of antioxidants as their pulp (118.50 µg/g dw) [13]. In addition, antioxidant levels peak when the fruit is ripe [10].

The present study was aimed at characterizing the metabolome by LC-ESI/LTQOrbitrap/MS analysis of the calyx and fruit to identify the majority of phytocompounds of *Physalis alkekengi* L. Subsequently, through preliminary spectrophotometric assays and then by cell studies, the antioxidant activity of various *Physalis alkekengi* L. extracts was evaluated. It was found that *Physalis alkekengi* L. extracts are a good source of metabolites with various biological activities, in particular, antioxidant activity capable of reducing the production of free radicals in the intestinal Caco-2 cell line.

## 2. Materials and Methods

### 2.1. Raw Materials

*P. alkekengi* L. was purchased online from Frutta Web (https://www.fruttaweb.com/) on 10 November 2019. The plant matrix was divided into two parts: leaves that comprise the calyx, and fruit. Two types of extraction were performed on these plant parts: decoction and hydroalcoholic extraction. On this basis, the samples were divided as follows: PACD (*P. alkekengi* L. calyx decoction), PAFD (*P. alkekengi* L. fruit decoction), PACE *(P. alkekengi* L. calyx hydroalcoholic extract), and PAFE (*P. alkekengi* L. fruit hydroalcoholic extract).

### 2.2. Reagents and Solvents

Ethanol (≥99.9%) and water (≥99.9%) employed for the extractions were bought from VWR (Milan, Italy). Acetonitrile (ACN), formic acid, water and methanol of LC-MS grade were purchased from Merk (Merk KGaA, Darmstadt, Germany). ABTS (2,20-azino-bis (3-ethylbenzothiazoline-6-sulfonic acid)), Trolox (6-hydroxy-2,5,7,8-tetramethylchroman-2-carboxylic acid), DPPH (2,2-Diphenyl-1-picrylhydrazyl), NaOH (sodium hydroxide), NaNO_2_ (sodium nitrite), AlCl_3_ (aluminum chloride), K_2_S_2_O_8_ (potassium persulfate), PBS (phosphate buffered saline), vitamin C and quercetin were purchased from Sigma-Aldrich (Milan, Italy).

2′,7′-dichlorodihydrofluoresceindiacetate (H_2_-DCF-DA) and MTT powder were purchased from Sigma Aldrich.

### 2.3. Sample Preparation

After the various parts of the *Physalis alkekengi* L. were divided, they were stored at −80 °C and then freeze-dried by a freeze dryer (LIO 5Pascal) that applied vacuum pressure and freezing temperature (−48 °C) to remove 95–98% of the water content of the plant materials while retaining their nutritional value and composition. The freeze-dried plant material was extracted using two extraction methods: by sonication with an ethanol/water solution (70:30) and by water decoction. For hydroalcoholic extractions, 1 g of freeze-dried matrix was used with 40 mL of ethanol/water solution (70/30) for the calyx, and 20 mL for the fruit. The extractions were performed in an ultrasonic bath (Grant, ultrasonic bath XUBA3) for 15 min at room temperature, repeating the extractions three times and then filtering the extracts with paper filters.

Amounts of 1 g of calyx and 2.5 g of fruit were used for decoctions with 50 mL of water, following EP pharmacopeia guidelines.

### 2.4. LC-ESI/LTQOrbitrap/MS and LC-ESI/LTQOrbitrap/MS/MS Analysis

A method was developed for metabolite profiling from decoctions and ethanolic extractions by coupling an HPLC with a hybrid mass spectrometer combining the linear trap quadrupole (LTQ) and the Orbitrap mass analyzer. Experiments were performed using a Thermo Scientific liquid chromatography system equipped with an Accela 600 quaternary pump, and an Accela autosampler coupled to a linear trap–Orbitrap hybrid mass spectrometer (LTQ-Orbitrap XL, Thermo Fisher Scientific, Bremen, Germany) with an electrospray ionization (ESI) source. A Phenomenex Luna C18 5 µm (150 mm × 2.00 mm) column (Phenomenex, Aschaffenburg, Germany) was used for the separation. The mobile phases used were water + 0.1% formic acid (A) and acetonitrile + 0.1% formic acid (B). An increasing linear gradient (*v*/*v*) at a flow rate of 0.200 mL/min of solvent B was used: 0–35 min, from 5 to 95%; 35–36 min, from 95 to 5%; and maintaining this percentage until 40 min. The experiments were performed in positive ion mode and 5 µL of each sample was injected. The parameters of the ESI source were as follows: capillary voltage, 49 V; tube lens voltage, 120 V; ion source temperature, 280 °C; sheath and auxiliary gas (N2) flow rates, 30 and 5; sweep gas, 0. The full m/z range suitable for recording MS spectra was 180–1600. To study the fragmentation pattern, a data-dependent scan was performed in which the precursor ions corresponding to the most intense peaks were fragmented in LC–MS analysis with a collision energy of 30%. Xcalibur software version 2.2 was used for instrument control, data acquisition and data analysis.

### 2.5. DPPH· Radical Scavenging Activity

The antioxidant activity of *Physalis alkekengi* L. was investigated by evaluating the anti-radical activity of the stables 1,1-diphenyl-2-picrylhydrazyl radical (DPPH·) according to the previously published method [14].

The first step was to prepare a methanolic solution of DPPH· with a concentration of 0.025 g/L. The samples to be studied were prepared by adding an aliquot (37.5 µL) of the methanolic solution containing different amounts of each extract (50, 100, and 200 µg/mL) to 1.5 mL of the previously prepared DPPH· solution.

The controls consisted of 37.5 µL and 1.5 mL of DPPH· solution. After 10 min, the absorbance was measured at 517 nm using a UV spectrophotometer (Spectrophotometer Multiskan Go, Thermo Scientific). All experiments were performed in triplicate.

The antioxidant extracts reduced the DPPH radical in the compound DPPH-H, resulting in a decrease in absorbance. Thus, the antiradical activity of the extracts was evaluated as a decrease in absorbance at 517 nm, more precisely expressed as a percentage of radical inhibition of DPPH according to Equation (1):

(1) $$\%\;Inhibition\;DPPH•=\frac{A_{0}-A_{e}}{A_{e}}*100$$

where A_0_ is the average of the absorbances of the control in triplicate and A_e_ is the average of the absorbances of each concentration of the various extracts in triplicate. Vitamin C was used as the reference compound.

### 2.6. Trolox Equivalent Antioxidant Capacity (TEAC) Assay

Trolox equivalent antioxidant capacity assay was set up as a further test following a protocol already published [14]. The extracts were diluted with water and methanol to obtain final concentrations of 250, 500, 750 and 1000 µg/mL. The assay was set up in 96-well plates, adding 15 µL of each sample to 150 µL of ABTS. The absorbance was measured at 734 nm. The experiments were performed in triplicate.

The percentage diminution in absorbance was calculated for each concentration relative to a blank absorbance (methanol/water) and plotted as a function of the concentration of the compound or standard 6-hydroxy-2,5,7,8-tetramethylchroman-2-carboxylic acid (Trolox).

The antioxidant activities were indicated as a TEAC value, i.e., the concentration of a Trolox standard solution with the same antioxidant activity as 1 mg/mL of the tested extract; quercetin-3-*O*-glucoside was used as the reference compound.

### 2.7. Cell Culture

The Caco-2 cell line was obtained from ECACC (Salisbury, UK). Caco-2 cells are derived from human colorectal adenocarcinoma which, once reaching confluence, spontaneously differentiate into normal enterocytes. Dulbecco’s modified Eagle’s medium (DMEM) with low glucose and with L-Arginin, phosphate-buffered saline (PBS) without MgCl_2_ and CaCl_2_, fetal bovine serum (FBS) and penicillin/streptomycin 1X were purchased from Euroclone (Milano, Italy). Caco-2 cells were grown in T75 flasks until their confluence reached 80%, at 37 °C in a 5% CO_2_ humidified atmosphere in DMEM supplemented with 10% FBS, and 1% antibiotic/antimycotic solution (100 U/mL penicillin, 100 mg/mL streptomycin) [15]. At passage 45–60, cells were removed from flasks by adding a trypsin solution at 1% and incubating at 37 °C for 5–10 min; Caco-2 cells were then collected, centrifuged and counted in a Bürker chamber and then seeded into 24- or 96-well plates at a concentration of 5 × 10^4^ cells/mL for different experiments. Cells were cultured replacing the medium twice weekly, to allow their spontaneous differentiation [16].

### 2.8. MTT Viability Test

To ascertain any cytotoxic activity of the phenolic extracts in normal differentiated (21 days post-seeding) cells and in intestinal cancer cells (three days post-seeding), Caco-2 cell viability was assessed using an MTT assay. The cells were seeded in 96-well plates (5 × 10^3^ cells/well in 100 μL) incubated with different concentrations (5–100 μg/mL) of methanolic extracts or to an equivalent volume of methanol for the controls, and were incubated for 24 h. At 24 h before treatment, the 10% serum supplemented medium was discarded and replaced with a medium with 2.5% serum. After incubation, the medium was removed and 100 μL of the MTT solution (5 mg/mL of MTT in PBS, 8%, in fresh serum free medium, 92%) was added and left for 4 h at 37 °C. The MTT solution was then discarded and 100 μL of DMSO was added to each well. Afterwards, the absorbance of each well was measured at 570 nm using a microplate reader (Infinite F200, Tecan, Salzburg, Austria). Cell survival was expressed as percentage of control (0 μg/mL) values.

### 2.9. Determination of Intracellular ROS Production

ROS production in Caco-2 cells was evaluated using the fluorescent probe H_2_-DCF-DA as reported by Gil et al. [17], with minor modifications. Cells were seeded in 96-well plates and once differentiated, were incubated with 10 μM of H_2_-DCF-DA in 100 μL of PBS for 30 min. Afterwards, H_2_-DCF-DA was replaced by the PBS solution containing the phenolic extract (5, 10, 25, 50 and 100 μg/mL), 30 min prior to adding tert-Butyl hydroperoxide (TBH) 2.5 mM to induce ROS release and lipid peroxidation. Control cells were treated only with PBS. The increase in cell fluorescence was determined using an Infinite F200 (Tecan, Salzburg, Austria) microplate reader at 485 and 530 nm (excitation and emission wavelengths, respectively). ROS production was monitored by reading the fluorescence emitted after 120 min of incubation. Moreover, cells treated with PAFE were analyzed as representative samples using a ZOE^TM^ fluorescent cell imager (Bio-Rad Laboratories, Hercules, CA, U.S.A.), using the green channel with a blue LED (excitation: 480/17 nm; emission: 517/23 nm) to the size of 100 μm.

### 2.10. Statistical Analyses

Data were analyzed by means of software GraphPad Prism 5 (GraphPad software, San Diego, CA, USA), using one-way analysis of variance (ANOVA) followed by post-hoc Tukey’s test. A level of *p* < 0.05 was considered statistically significant.

## 3. Results

### 3.1. Identification of Metabolites in Physalis alkekengi L. Fruit and Calyx Extracts by LC-ESI/LTQOrbitrap/MS and LC-ESI-LTQOrbitrap/MS/MS Analysis

**Figure 1:**
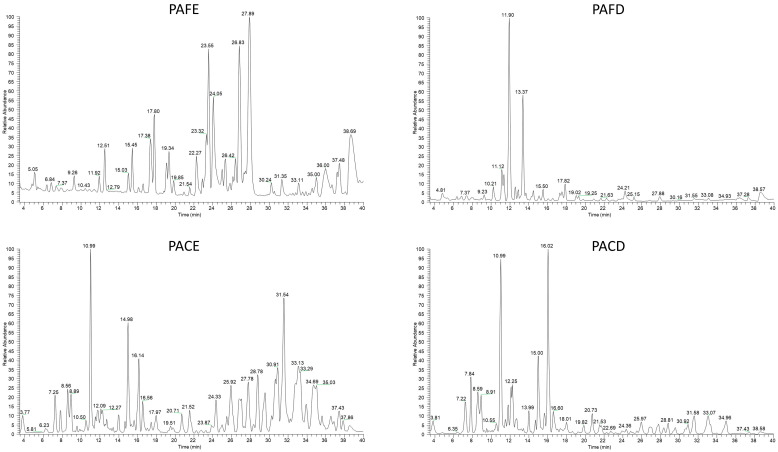
Positive ion mode profiles of *Physalis alkekengi* L. ethanolic extracts obtained by LC-ESI/LTQ/Orbitrap MS: PAFE (*Physalis alkekengi* L. fruit ethanolic extraction), PACE (*Physalis alkekengi* L. calyx ethanolic extraction), PAFD (*Physalis alkekengi* L. fruit decoction), and PACD (*Physalis alkekengi* L. calyx decoction).

Metabolite profiles of hydroalcoholic and decoction extracts obtained from *Physalis alkekengi* L. fruit and calyx analyzed by LC-ESI-LTQ-MS/MS and investigated by Xcalibur Software, and the total ion current LC-MS chromatograms for the two different extraction methods applied to the two different parts of the plant under investigation, are reported in Figure 1. Accurate mass measurement (ppm ≤ 5) and fragmentation experiments (MS/MS) obtained under data dependent scan mode, associated with research in specific databases on spectral data for natural substances as KNApSAcK (www.knapsackfamily.com) on 12 November 2023 and in the literature for the species *Physalis alkekengi* L., allowed the putative identification of 58 secondary metabolites mainly belonging to flavonoid glycosides, steroids, phenolic glycosides, coumarins, phenolic acids, carotenoids, and N-containing compounds (Table 1). Flavonoids were among the major components of *Physalis alkekengi* L., with a common C6–C3–C6 tricyclic skeleton [18]. Compounds **1**, **7**, **11**, **16**, **18, 19**, **21**, **23**, **24** were identified as flavonoid glycosides, occurring both in the fruit and calyx of *Physalis alkekengi* L., and were observed as the major compounds when the extraction method was decoction. Flavonoid aglycons quercetin (**37**) and luteolin (**41**) were recovered in fruit and calyx by using both extraction methods, while apigenin (**50**) was identified in the calyx and not the fruit. The interesting presence of flavonoids in the fruit and calyx of *Physalis alkekengi* L. was reviewed in 2018 by Li et al. [6].

In addition to the presence of organic acids, *p*-coumaric acid (**2**), quinic acid (**6**), 1,3-dicaffeoylquinic acid (**9**), 4,5-dicaffeoylquinic acid (**12**), 3-caffeoylquinic acid (**22**), 5-caffeoylquinic acid (**43**), and caffeic acid (**25**) were revealed together with their derivatives, as suberic acid (**4**), feruloyl dihexoside (**5**), feruloyl hexoside (**32**), caffeoylglycerol (**36**), and coumaroyl dihexoside (2 isomers, **45** and **46**). Coumaroyl and feruloyl glycosides largely contribute to the antioxidant activity of diverse plant materials [19].

The presence of these compounds is probably involved in the antioxidant activity of the calyx and fruit of *Physalis alkekengi* L. In addition, in the *Physalis alkekengi* L. fruit and calyx, in agreement with Wen and co-authors [20], these compounds were found in the final part of the chromatograms of hydroalcoholic extracts compounds identified as carotenoids (**52**, **53**, **54**, **55**, **56**, **57**, **58**). The same compounds were not identified in the decoction extracts, due to the polarity of the extraction solvent (water).

**Table 1:** Metabolite profiling of yellow *Physalys alkekengi* L. fruit and calyx by LC-ESI-LTQ-Orbitrap-MS/MS. PACE (*Physalis alkekengi* L. calyx hydroalcoholic extract), PAFE (*Physalis alkekengi* L. fruit hydroalcoholic extract) PACD (*Physalis alkekengi* L. calyx decoction extract), PAFD (*Physalis alkekengi* L. fruit decoction extract).

N°	Rt	Identity	Molecular Formula	[M + H]^+^	Δppm	MS/MS	References	PACE	PAFE	PACD	PAFD
**1**	7.81	quercetin 3-*O*-rutinoside-7-*O*-glucoside	C_33_H_40_O_20_	773.2162	3.5	611; 449; 303;	Mass Bank	nd	nd	x	x
**2**	8.56	*p*-coumaric acid	C_9_H_6_O_3_	163.0391	−2.5	165; 147	[21]	nd	nd	x	x
**3**	8.57	hydroxycoumarin	C_9_H_6_O_3_	163.0395	0.9	163; 145	[21]	x	x	x	x
**4**	8.92	suberic acid	C_8_H_13_O_4_	175.0816	−1.8	157; 139	[21]	nd	nd	x	x
**5**	9.15	feruloyl dihexoside (2)	C_22_H_30_O_14_	519.1713	0.7	195	[22]	x	x	x	x
**6**	9.25	quinic acid	C_7_H_12_O_6_	193.0556	2.1	135; 117	[21]	x	x	x	x
**7**	10.96	rutin	C_27_H_30_O_16_	611.1534	−0.6	465; 303	[23]	x	x	x	x
**8**	11.26	5,6*α*-epoxy-physalin C	C_28_H_30_O_10_	527.1937	−1.5	509; 499; 483	[21]	x	nd	x	nd
**9**	11.49	1,3-dicaffeoylquinic acid	C_25_H_24_O_12_	517.1344	0.6	193	[21]	x	x	x	x
**10**	11.68	4-hydroxyneophysalin A isomer	C_28_H_30_O_11_	543.1866	−0.5	511; 497; 449	[21]	x	nd	nd	nd
**11**	11.82	luteolin-rutinoside	C_27_H_30_O_15_	595.1584	−0.7	449; 287	Mass Bank	x	x	x	x
**12**	12.66	4,5-dicaffeoylquinic acid	C_25_H_24_O_12_	517.1344	1	284; 269; 141	[21]	x	x	x	x
**13**	13.13	L-tryptophan	C_11_H_12_N_2_O_2_	205.0707	2.3	171; 147	[22]	x	x	x	x
**14**	14.91	4-hydroxyneophysalin A isomer	C_28_H_29_O_11_	541.1713	−1	513; 497; 465	[21]	x	nd	x	nd
**15**	15.59	4-hydroxyneophysalin A	C_28_H_30_O_11_	541.1866	0.5	513; 497; 465	[21]	x	x	x	x
**16**	15.95	isolariciresinol *O*-glucoside	C_26_H_34_O_11_	523.2179	1	505; 184	[21]	x	x	x	x
**17**	15.96	5,6-O-epoxy-physalin C isomer	C_28_H_30_O_10_	527.1937	−1.5	509; 499; 483	[21]	x	nd	x	x
**18**	16.14	quercetin 3-*O*-glucoside	C_21_H_20_O_12_	465.1033	−0.9	303; 285	[21]	x	x	x	x
**19**	16.67	luteolin 7-*O*-glucoside	C_21_H_20_O_11_	449.1083	−0.9	287; 285	[21]	x	x	x	x
**20**	16.91	5,6-*O*-epoxy-physalin C	C_28_H_30_O_10_	527.1917	0.8	509; 499; 483	[21]	x	nd	x	nd
**21**	16.99	quercetin *O*-rhamnoside	C_21_H_20_O_11_	449.1083	−0.9	303; 287	[21]	x	x	x	x
**22**	17.33	3-caffeoylquinic acid	C_16_H_18_O_9_	355.0872	0.5	337; 284	[21]	x	x	x	x
**23**	17.92	vitexin	C_21_H_20_O_10_	433.1134	1.2	433; 417; 379	[21]	x	x	x	x
**24**	17.92	apigenin *O*-glucoside	C_21_H_20_O_10_	433.1134	1.2	271	[21]	x	x	x	x
**25**	17.94	caffeic acid	C_9_H_8_O_4_	181.0500	−2.2	163	[21]	x	x	x	x
**26**	18.21	4-hydroxyneophysalin A isomer	C_28_H_30_O_11_	541.1866	0.8	284; 198; 141	[21]	x	x	x	x
**27**	19.82	4,7-didehydro-7-deoxyneophysalin A	C_28_H_28_O_9_	509.1811	0.7	497; 465	[21]	x	x	x	nd
**28**	19.82	N,N′-dicaffeoylspermidine (1)	C_25_H_31_N_3_O_6_	470.2211	0.5	333; 289	[22]	x	x	x	x
**29**	19.86	5-ethoxy-6-hydroxy-5,6-dihydrophysalin B	C_30_H_36_O_11_	573.2335	0.7	555; 523	[21]	x	nd	x	nd
**30**	19.97	physalin Z	C_28_H_30_O_10_	527.1917	0.6	509; 499; 483	[21]	x	x	x	x
**31**	19.97	physalin A	C_28_H_30_O_10_	527.1917	0.8	509; 499; 483	[21]	x	x	x	x
**32**	19.97	feruloyl hexoside	C_16_H_20_O_9_	355.1040	1.8	195; 179	[22]	x	x	x	x
**33**	20.71	physalin C	C_28_H_30_O_9_	511.1968	0.6	493; 481; 423	[21]	x	x	x	x
**34**	20.73	physalin O	C_28_H_32_O_10_	529.1917	0.4	511; 483	[21]	nd	nd	x	nd
**35**	21.85	physalin N	C_28_H_30_O_10_	527.1917	0.6	509; 499; 483	[21]	x	x	x	x
**36**	21.87	caffeoylglycerol	C_12_H_14_O_6_	255.0712	0.8	214	[21]	nd	x	nd	x
**37**	21.9	quercetin	C_15_H_10_O_7_	302.2303	0.7	257; 229	Mass Bank				
**38**	21.99	isophysalin B	C_28_H_30_O_9_	511.1968	0.6	493; 467; 423	[21]	x	x	x	nd
**39**	23.28	N,N′-dicaffeoylspermidine (2)	C_25_H_31_N_3_O_6_	470.2211	0.5	333; 289	[22]	x	x	x	x
**40**	23.55	sonchuionoside C	C_19_H_30_O_8_	387.2018	−0.7	331; 198	[21]	x	x	x	x
**41**	23.9	luteolin	C_15_H_10_O_6_	285.0383	1.3	153; 135	[21]	x	x	x	x
**42**	23.91	4,7-didehydro-physalin B	C_28_H_28_O_9_	509.1811	0.7	497; 465	[21]	x	x	x	nd
**43**	24.02	5-caffeoylquinic acid	C_16_H_18_O_9_	355.0871	1.5	198; 141; 109	[21]	x	x	x	x
**44**	24.33	7β-hydroxy-25,27-didehydrophysalin L	C_28_H_30_O_10_	527.1917	0.6	509; 499; 483	[21]	x	x	x	x
**45**	24.81	coumaroyl dihexoside	C_21_H_28_O_13_	489.1608	1.7	327	[22]	x	x	x	x
**46**	25.46	coumaroyl hexoside	C_15_H_18_O_8_	327.1079	2.1	188; 165	[22]	x	x	x	x
**47**	26.14	4,7-didehydrophysalin B	C_28_H_28_O_9_	509.1811	0.7	509; 499; 483	[21]	x	x	x	nd
**48**	26.41	physalin M	C_28_H_32_O_9_	513.2124	0.8	495; 451; 135	[21]	x	nd	x	x
**49**	26.82	4,7-didehydroneophysalin B	C_28_H_28_O_9_	509.1811	0.7	497; 465	[21]	x	x	x	nd
**50**	28.41	apigenin	C_15_H_10_O_5_	271.0606	1.6	153	[21]	x	nd	x	nd
**51**	28.45	physalin B	C_28_H_30_O_9_	511.1968	0.6	493; 467; 423	[21]	x	x	x	nd
**52**	30.63	(all-E)-antheraxanthin	C_40_H_56_O_3_	585.4307	1.3	567; 549; 493	[20]	x	x	nd	nd
**53**	30.91	zeaxanthin myristate	C_54_H_82_O_3_	779.6342	0.6	761; 687; 669; 551; 533	[20]	x	x	nd	nd
**54**	31.18	(13Z)-β-carotene	C_40_H_56_	537.4460	1.3	445; 347; 281; 255	[20]	x	x	nd	nd
**55**	31.18	(all-E)-α-carotene	C_40_H_56_	537.4460	1.3	481; 399	[20]	x	x	nd	nd
**56**	31.46	(all-E)-lutein	C_40_H_56_O_2_	569.4358	1.4	551	[20]	x	x	nd	nd
**57**	32.49	(15Z)-zeaxanthin	C_40_H_56_O_2_	569.4358	1.4	551	[20]	x	x	nd	nd
**58**	33.96	(all-E)-zeaxanthin	C_40_H_56_O_2_	569.4358	1.4	551	[20]	x	x	nd	nd

nd: not determined.

Metabolites characteristic of the genus *Physalis* are a group of steroids bearing 13, 14-seco-16,24-cycloergostane skeletons, named as physalins. Physalin A (**31**), isolated from the leaves of *P. alkekengi* var. franchetii in 1969, was the first member of this group [24]. Subsequently, a series of physalins with complex and diverse structures have been reported from the genus *Physalis* [6,25,26].

In recent decades, about fifty physalins have been identified and reported to exert anti-inflammatory, antimicrobial, anti-diabetes, cytotoxic, and quinone reductase induction activities [27,28].

The present metabolomics approach allowed the putative identification of 21 compounds pertaining to this group of metabolites (**8**, **10**, **14**, **15**, **17**, **20**, **26**, **27**, **29**, **30**, **31**, **33**, **34**, **35**, **38**, **42**, **44**, **47, 48**, **49**, **51**), showing that these compounds have the ability to give good [M + H]^+^ ions for LC-MS identification. Until now they have been identified mainly by LC-MS in negative ionization mode [25].

Other identified compounds in the metabolite profiling approach were metabolites pertaining to various different classes: coumarins (**3**), amino acids (**13**), lignans (**16**), nitrogen containing compounds (**39**), terpene glycosides (**40**); all compounds that have been reported previously in genus *Physalis* [6].

Although a quantitative targeted approach was not performed due to the unavailability of standard compounds, by visual comparison of profiles it was possible to note substancial differences in the fruit and calyx profiles, but even more evident were differences in extracts obtained with different extraction modes. Observing Figure 1, it is easy to evaluate that in decoctions, the most evident metabolites were those eluted at retention times between 4 and 18 min. Comparing this evidence with the table of characterization, these compounds were phenolic acids and flavonoids. On the other hand, physalins start to be eluted after 18 min. Physalins were identified in the decocted extract too, but in very low amounts. This finding means that decoction is an extraction method that permits an extract enriched in polyphenols—which are highly involved in antioxidant activity—with a lower amount of potentially toxic physalins.

### 3.2. Antioxidant Activity Evaluated by Spectrophotometric Assays

Plants of the genus *Physalis* are widely used as medicinal plants for the treatment of asthma, dermatitis, and rheumatism, and to prevent cancer [29]. Given the high content of compounds with antioxidant activity such as physalins and glicosylated flavonoids, the antioxidant activities of *Physalis alkekengi* L. extracts were investigated by spectrophotometric assays (Table 2).

The *Physalis alkekengi* L. extracts tested showed good antioxidant activity, regardless of the type of extraction performed, confirming that both hydroalcoholic extraction and decoction allow the extraction of compounds with antioxidant activity. Data obtained for the samples were compared with the positive controls, quercetin 3-*O*-glucoside for TEAC assay and vitamin C for DPPH assay. However, the decoctions were slightly more active than the hydroalcoholic extracts, possibly due to a higher content of extracted glycosylated flavonoids, as indicated by the metabolite profiling performed on the samples.

On the other hand, as far as the analyzed part of the plant is concerned, the calyx extracts were certainly more active than those derived from fruit. In fact, calyx extracts reached TEAC values (equivalent antioxidant capacity in Trolox expressed in mg/mL) of 2.382 for PACD and 1.749 for PACE and, respectively, an IC_50_ for DPPH (expressed in mg/mL) of 0.017 for PACD and 0.033 for PACE.

The finding was in agreement with antioxidant activity measured for *Physalis alkekengi* fruit extract by Vicas et al. (2020) by DPPH test, and Liu et al. (2023) by ABTS and DPPH chemical tests [30,31].

### 3.3. Cell Viability

**Figure 2:**
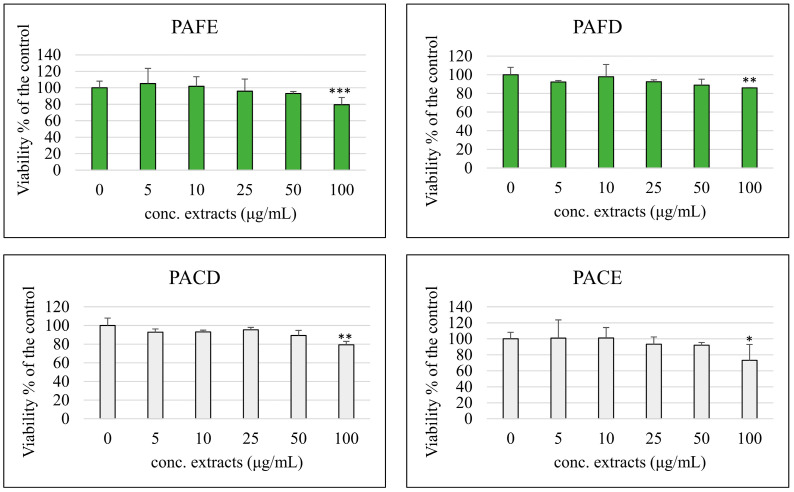
Percentage of cell viability compared with the control (0 μg/mL, 100% viable cells) of differentiated Caco-2 cells incubated for 48 h with different concentrations (5–100 μg/mL) of phenolic extracts derived from fruit extract (PAFE), fruit decoction (PAFD), calyx extract (PACE) and calyx decoction (PACD). Each column represents the mean ± SD of the independent experiments (*n* = 16). *** = *p* < 0.001 vs. 0 μg/mL; ** = *p* < 0.01 vs. 0 μg/mL; * = *p* < 0.05 vs. 0 μg/mL.

**Figure 3:**
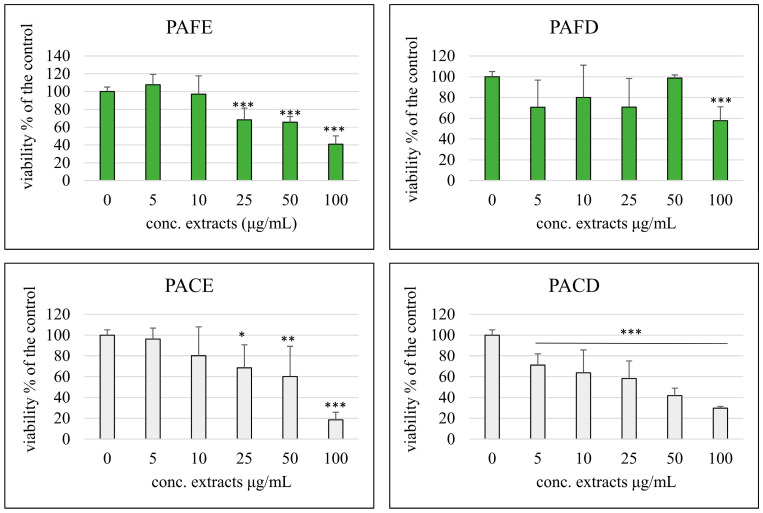
Percentage of cell viability compared with the control (0 μg/mL, 100% viable cells) of cancer Caco-2 cells incubated for 48 h with different concentrations (5–100 μg/mL) of phenolic extracts derived from fruit extract (PAFE), fruit decoction (PAFD), calyx extract (PACE) and calyx decoction (PACD). Each column represents the mean ± SD of the independent experiments (*n* = 16). *** = *p* < 0.001 vs. 0 μg/mL; ** = *p* < 0.01 vs. 0 μg/mL; * = *p* < 0.05 vs. 0 μg/mL.

The possible cytotoxic effect of the four extracts was assessed on cancer or normal differentiated Caco-2 cells at concentrations ranging from 5 to 100 μg/mL using the MTT assay. Figure 2 summarizes the cell viability results on differentiated cells. As shown, the tested extracts did not cause a decrease in the cellular viability of Caco-2 cells, showing results of around 100% of the control (0 μL extract/mL) with the exception of the concentration 100 μg/mL, which resulted as being significantly cytotoxic for all four extracts. The same treatment was also carried out on non-differentiated cancer cells, to explore the anti-tumoral and anti-proliferative potential of these extracts (Figure 3).

It was observed how extracts derived from the *Physalis alkekengi* L. fruit extract and decoction were significantly cytotoxic in Caco-2 starting from 25 μg/mL, while the fruit decoction extract was toxic only at the highest concentration tested. Among the extracts tested, the most effective in exerting toxic effects on Caco-2 cells was the one derived from calyx decoction, which was found to be significantly toxic starting from the minimum concentration tested (5 μg/mL). Given the results obtained in normal cells, we decided to use 50 μg/mL as the maximum concentration, being the the highest non-toxic one, in the subsequent experiments.

### 3.4. ROS Production

**Figure 4:**
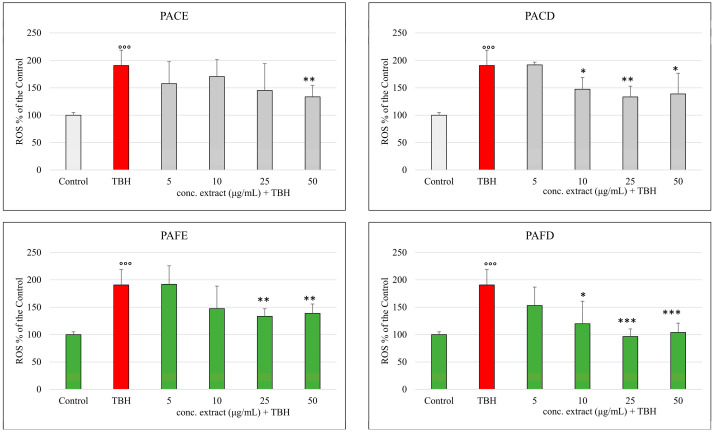
Determination of intracellular ROS production. ROS level, visualized as H2-DCF-DA fluorescence and expressed as a % of the control samples (non-oxidized nor pretreated samples), in Caco-2 after 120 min incubation with TBH 2.5 mM and pretreated with the four extracts (5–50 μg/mL). °°° = *p* < 0.001 vs. control; *** = *p* < 0.001 vs. TBH 2.5 mM; ** = *p* < 0.01 vs. TBH 2.5 mM; * = *p* < 0.05 vs. TBH 2.5 mM.

The ability of the four different extracts to scavenge reactive species was also tested in Caco-2 cell cultures (Figure 4).

**Figure 5:**
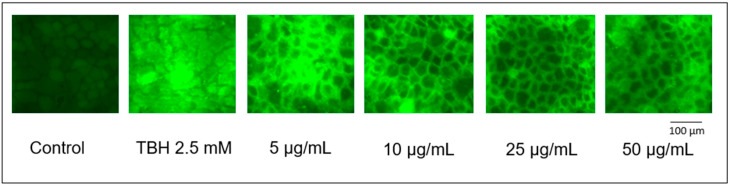
ROS levels visualized as H2-DCF-DA fluorescence in differentiated Caco-2 after 30 min incubation with TBH 2.5 mM and pretreated with the phenolic extract from PAFE (30 min).

Determination of intracellular ROS production and subsequent oxidative damage to cell membranes was induced by TBH at the concentration of 2.5 mM. In cells pretreated with all four phenolic extracts, inhibition of ROS formation was observed, as indicated by the lower emission of fluorescence. This inhibition was observed starting from a concentration of 10 μg/mL in the extracts derived from the *Physalis alkekengi* L. fruit (*p* < 0.05), while in those derived from calyces, minimum concentrations of 25 μg/mL for the decoction and 50 μg/mL for the extract were needed to observe a significant inhibition against TBH-induced peroxidation. To better highlight the antioxidant action of the extracts, we captured as a representative example the fluorescence emitted by cells treated with fruit extract by ZOE^TM^, as can be observed in Figure 5, in which it is possible to more clearly see the inhibition of ROS production.

## 4. Discussion

The fruit and calyx from *Physalis alkekengi* L. are considered to be the only edible parts of the plants. The interesting results show the possibility of using the fruit and calyx for the preparation of tea infusion. To date, a few chemical investigations have been performed on this cultivar, mainly focusing on the analysis of methanol extracts. Due to the increasing use of *Physalis* as a food supplement and starting material for tea preparation, in this study a comprehensive analysis of green extracts was performed.

The comprehensive metabolomics analysis performed by LC-LTQ-Orbitrap-MS and LC-LTQ-Orbitrap-MS/MS showed no relevant differences in the composition of calyx and fruit. On the other hand, the extracts obtained by decoction showed some differences with respect to EtOH:H_2_O extracts: in fact, they presented the same composition with regard to flavonoids, phenylpropanoids, organic acids, but were characterized by a low amount of physalins, and they were not characterized by the presence of carotenoids, identified in hydroalcoholic extracts. Previous studies have found that carotenoids in *Physalis alkekengi* L. fruit have good antioxidant effects and safety [32].

The metabolomics approach allowed the identification of 58 compounds confirming the data reported in different previous phytochemical studies, extensively reviewed in 2018 by Li et al. [6].

Comparison of antioxidant activity of calyx and fruit extracts shown in the preliminary chemical tests revealed higher activity for the calyx.

Despite the absence of carotenoids, calyx extract obtained by decoction showed a higher antioxidant activity in both TEAC and DPPH assays, due to the presence of a higher content of flavonoid glycosides; in fact, reducing and free radical scavenging abilities are always positively correlated with total flavonoids content.

The cytotoxicity studies on intestinal Caco-2 cancer cells showed a difference between fruit and calyx, with major toxicity induced by calyx extracts and significantly less toxicity for extracts obtained by decoction. This may be due to the different composition in compounds that may exert higher cytotoxic effects or can be better absorbed by intestinal cancer cells [33]. It is notable that this toxicity towards cancer cells did not occur, with the exception of the highest concentration (100 µg/mL), in normal intestinal cells, suggesting a safety profile in regard to all extracts tested.

Regarding the inhibition of ROS production, in addition to the better activity of the extracts obtained by decoction with respect to the hydroalcoholic extracts, we observed the best activity for decoction extract when derived from fruit. In this specific case, we can speculate that probably in fruit, in addition to flavonoids, we may find the presence of polysaccharides, that participate in the scavenging capacity with synergistic effect when the antioxidant activity is measured on cells [34]. The activity carried out by the antioxidant compounds present in *Physalis alkekengi* L. extracts may merely be a free radical scavenging action, but also, modulatory effects towards redox-sensitive pathways which regulate the redox state of the cell may occur [35]. In particular, it has been widely observed how some polyphenols such as quercetin or cinnamic acid derivatives are able to modulate at intestinal level the MAP kinases signaling pathway and regulate the expression of Nrf-2, a transcription factor that induces the expression of antioxidant enzymes such as heme oxygenase (HO)-1, NAD(P)H:quinone oxidoreductase (NQO1), superoxide dismutase (SOD), and glutathione peroxidase (GSH-Px) [29,36]. Furthermore, it must be considered that many of the compounds present in the extracts can easily be absorbed and metabolized by intestinal cells, forming a pool of equally active phase I/II metabolites. These metabolites can act as primary antioxidants, but are also able to modulate signaling pathways and induce the expression of antioxidant enzymes [37].

In particular, physalins are characterized by fast absorption, wide distribution, and rapid excretion. Our findings indicated that physalins are extremely unstable and have low bioavailability in the intestine, which reduces their long-term toxicity. On the other hand, it should be noted that few studies have investigated the pharmacokinetics of extracts and active compounds involved in antioxidant activity, i.e., flavonoids. Consequently, further pharmacokinetics studies in the laboratory and clinic analyses should be performed for a comprehensive approach to the use of *Physalis alkekengi* for its antioxidant effect in therapy [7].

## 5. Conclusions

An LC-ESI-LTQ/OrbitrapMS and LC-ESI-LTQ/OrbitrapMS/MS metabolomics approach allowed the characterization of metabolites present in different parts of *Physalis alkekengi* L. The preparation of two different types of extraction—hydroalcoholic extraction and decoction—showed that the highest detection of metabolites was obtained through hydroethanol extraction, with the putative identification of 58 metabolites. Subsequently, through preliminary spectrophotometric assays and then with cell studies, the antioxidant activity of various *Physalis alkekengi* L. extracts was evaluated. It was found that *Physalis alkekengi* L. extracts are a good source of metabolites with various biological activities, in particular, antioxidant activity capable of reducing the production of free radicals in Caco-2 cell-line.

An interesting result was the antioxidant activity of extracts obtained by decoction, that allows the suggestion of a potential beneficial use of *Physalis alkekengi* L. fruit and calyx in the preparation of tea infusions.

The innovative aspect of the present study was connected with the preparation of green extracts. For the first time, an integrated approach (metabolomics approach and antioxidant evaluation) was applied to the study of *Physalys alkekengi*’s decoctions, the most often used green extraction method in herbal preparations.

## Figures and Tables

**Table 2:** Antioxidant activity of extracts of *Physalis alkekengi* L. evaluated by Trolox equivalent antioxidant capacity (TEAC) and DPPH• radical scavenging activity assays.

*Physalis alkekengi* L. Extracts	TEAC [mg/mL ± SD^a^] ^b^	DPPH [IC_50_ (mg/mL) ± SD^a^]
PACD	2.382 ± 0.020	0.017 ± 0.003
PAFD	0.720 ± 0.004	0.502 ± 0.010
PACE	1.749 ± 0.010	0.033 ± 0.003
PAFE	0.731 ± 0.002	0.670 ± 0.020
Quercetin 3-*O*-glucoside	2.426 ± 0.010	/
Vitamin C	/	0.170 ± 0.020

SD^a^, standard deviation of three independent experiments; ^b^, antioxidant activity determined by TEAC assay, and expressed as antioxidant capacity equivalent in Trolox in mg/mL. PAFE (*Physalis alkekengi* L. fruit ethanolic extraction), PACE (*Physalis alkekengi* L. calyx ethanolic extraction), PAFD (*Physalis alkekengi* L. fruit decoction), and PACD (*Physalis alkekengi* L. calyx decoction).

## Data Availability

The data used to support the findings of this study can be made available by the corresponding author upon request.
